# Serum markers change for intraocular metastasis in renal cell carcinoma

**DOI:** 10.1042/BSR20203116

**Published:** 2021-09-13

**Authors:** Tie Sun, Jing Tang, Yi-Cong Pan, Chen-Yu Yu, Biao Li, Li-Juan Zhang, Hui-Ye Shu, Qian-Min Ge, Yi Shao

**Affiliations:** 1 Department of Ophthalmology, The First Affiliated Hospital of Nanchang University, Nanchang 330006, Jiangxi Province, China; 2 The First Clinical Medical College, Nanchang University, Nanchang 330006, Jiangxi Province, China; 3 Department of Oncology, The Affiliated Zhuzhou Hospital Xiangya Medical College CSU, Zhuzhou 412000, Hunan, China

**Keywords:** intraocular metastasis, NSE, renal cell carcinoma, risk factors

## Abstract

**Objective:** Renal cell carcinoma is prone to early metastasis. In general, intraocular metastasis (IOM) is not common. In the present study, we studied the relationship between different biochemical indicators and the occurrence of IOM in renal cancer patients, and identified the potential risk factors.

**Methods:** A retrospective analysis of the clinical data of 214 patients with renal cell carcinoma from October 2001 to August 2016 was carried out. The difference and correlation of various indicators between the two groups with or without IOM was analyzed, and binary logistic regression analysis was used to explore the risk factors of IOM in renal cancer patients. The diagnostic value of each independent related factor was calculated according to the receiver operating curve (ROC).

**Results:** The level of neuron-specific enolase (NSE) in renal cell carcinoma patients with IOM was significantly higher than that in patients without IOM (*P*<0.05). There was no significant difference in alkaline phosphatase (ALP), hemoglobin (Hb), serum calcium concentration, α fetoprotein (AFP), carcinoembryonic antigen (CEA), CA-125 etc. between IOM group and non-IOM (NIOM) group (*P*>0.05). Binary logistic regression analysis showed that NSE was an independent risk factor for IOM in renal cell carcinoma patients (*P*<0.05). ROC curve shows that the factor has high accuracy in predicting IOM, and the area under the curve (AUC) is 0.774. The cut-off value of NSE was 49.5 U/l, the sensitivity was 72.2% and the specificity was 80.1%.

**Conclusion:** NSE concentration is a risk factor for IOM in patients with renal cell cancer. If the concentration of NSE in the patient’s body is ≥49.5 U/l, disease monitoring and eye scans should be strengthened.

## Introduction

Renal cell carcinoma accounts for approximately 3% of adult malignant tumors and is a common tumor of urinary system, the total incidence is approximately 3.39 [1]. In recent years, the incidence rate is increasing year by year. According to the pathological classification, there are more than ten subtypes of renal cell carcinoma, of which clear cell carcinoma is the main pathological type [2]. The course of renal cell carcinoma is insidious, and 20–30% of patients have metastasis at the time of initial diagnosis [3]. Once renal cell carcinoma metastasizes, its prognosis is not optimistic. The 5-year survival rate is less than 10%, and the median survival time is only 13 months [4]. The common way of metastasis of renal cell carcinoma is through the vein or lymphatic system. Renal cell carcinoma often invades the veins, spreading to the lungs and then to other tissues. The most prone to distant metastasis is the lung, which accounts for approximately 45%. The second is bone metastasis, which accounts for approximately 30%. The liver is closely followed, accounting for approximately 20%. Then there are the adrenal glands, which account for 9%, and the brain, which accounts for 8% [5]. In the lymphatic system, metastasis was first found in the renal hilar lymph nodes or paraaortic or paranasal lymph nodes. More than 50% of renal cell carcinoma patients had no obvious clinical symptoms [6]. And intraocular metastasis (IOM) often occurs in the late stage of renal cell carcinoma, which is relatively rare [7]. Therefore, the IOM of renal cell carcinoma is rarely reported in the literature. At present, the common diagnostic methods of renal cell carcinoma are CT, ultrasound and MRI. In ultrasound examination, patients with renal cell carcinoma are often accidentally found, and the diagnosis mainly depends on CT or MRI. CT is the most commonly used detection method because of its low cost and high operability. However, it is not enough to rely on imaging detection alone, because they have a series of limitations, such as high detection cost, easy to miss diagnosis, misdiagnosis and continuous multiple uses may lead to high-dose radiation. Moreover, it is impossible to conduct a comprehensive and detailed examination of some early IOMs [8]. To improve the prognosis of patients, it is necessary to detect and diagnose the abnormalities in patients as soon as possible. With the development of serological diagnosis technology, tumor markers detection has gradually become the most important auxiliary diagnostic method for urinary system tumors in clinical application. It is an ideal method for early diagnosis of urinary system tumors. It is very helpful for the early diagnosis of IOM, the risk factors of clinical significance, and the treatment and prognosis of renal cell carcinoma. Some serum tumor markers, such as NSE, are considered to be an important diagnostic and prognostic indicator for patients with renal cell carcinoma. The abnormal expression of NSE is a risk factor for many tumor diseases, and it is closely related to the disease stage and prognosis [9]. Nevertheless, the predictive and diagnostic value of NSE for IOM in renal cell carcinoma patients is still unclear, and the results are still controversial. The present study hopes to explore the relationship between serum tumor markers, clinicopathological parameters and the occurrence of IOM in patients with renal cell carcinoma, and to identify independent risk factors for IOM in patients with renal cell carcinoma.

## Materials and methods

### Patient selection

The Medical Research Ethics Committee of the First Affiliated Hospital of Nanchang University approved the present study, and the study was conducted by the ‘Declaration of Helsinki’ and related guidelines and regulations. The study samples are a series of patients diagnosed with kidney cancer in our hospital from October 2001 to August 2016. The diagnosis is made based on pathological specimens obtained through surgical resection or biopsy. The diagnosis of IOM is based on CT and MRI scans. Each participant received detailed information about this design study and signed an informed consent form related to the study.

### Data collection

The demographic and clinical characteristics of the patients in the present study included: patient’s age, gender, primary cancer location, pathological type, presence or absence of metastasis, metastatic site, serological data were collected from both groups of subjects, such as alkaline phosphatase (ALP), calcium and hemoglobin (Hb) levels, common tumor markers (serum NSE, α fetoprotein (AFP), carcinoembryonic antigen (CEA), CA-125, CA-153 and CA-199 values). We collected and reviewed the above factors. The correlation between the clinical indicators and IOM was analyzed, and the clinical characteristics were evaluated, and the risk factors of IOM were determined.

### Statistical analysis

The differences and clinical features between the IOM group and the non-IOM (NIOM) group were analyzed by Student’s *t* test and the Chi-square test. A binary logistic regression model was used to analyze the independent risk factors of IOM in renal cell carcinoma. Receiver operating curve (ROC) curve was established and area under the curve (AUC) was calculated to evaluate the accuracy of IOM prediction. The specificity and sensitivity were calculated by the critical value. *P*<0.05, indicating that the analysis results were statistically significant. Using IBM SPSS version 17.0 software (SPSS, Chicago, Illinois, U.S.A.) to conduct all relevant analyses in the study. The mean value ± standard deviation is used to represent continuous data.

## Results

### Patient demographics and clinical characteristics

**Figure 1:**
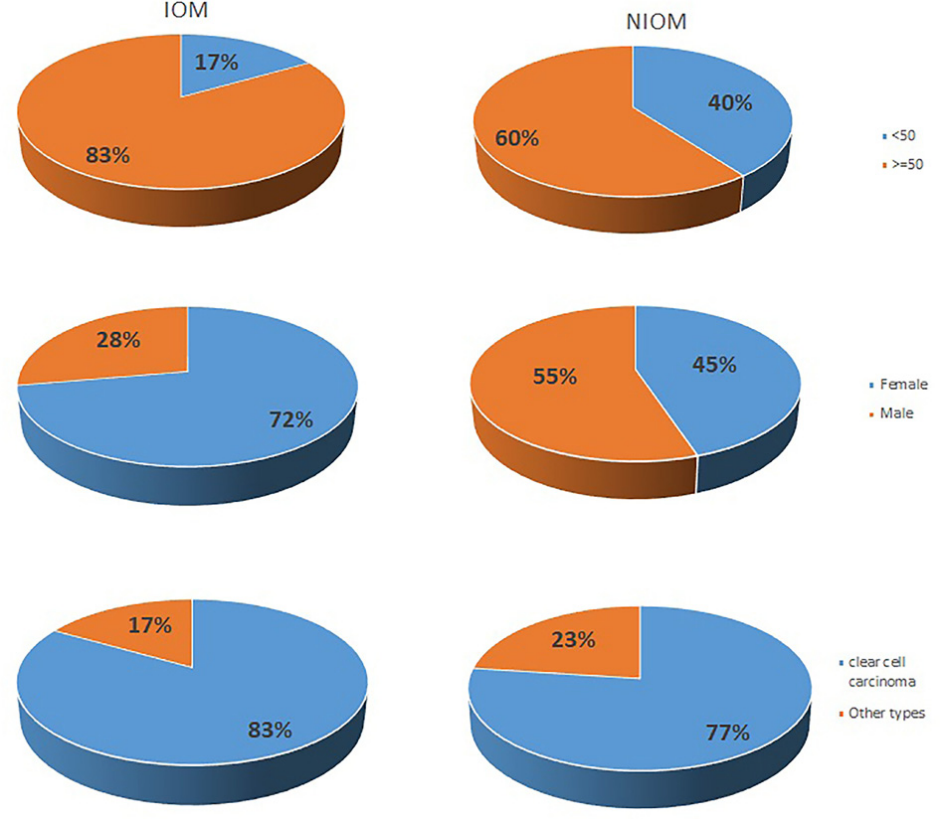
The clinical characteristics of renal cell cancer IOM and NIOM patients

A total of 214 renal cell carcinoma patients participated in the study, including male and female patients. There were 18 patients in the IOM group and 196 in the NIOM group. The mean age of IOM group in patients with renal cell carcinoma was 59.83 ± 12.21 years, and the mean age of NIOM group in patients with renal cell carcinoma was 55.71 ± 15.59 years. Among these renal cell carcinoma patients, there were 113 males and 101 females. Most of the histopathological types were clear cell carcinoma (166 cases, 77.6%). Of the patients with IOM, 15 were clear cell carcinoma and 3 were other types. Among patients with NIOM, 151 were clear cell carcinoma and 45 were other types (Table 1 and Figure 1).

**Table 1:** Baseline characteristics of patients with renal cancer

	IOM group (%)	NIOM group (%)	Total numbers of patients (%)	*P*-value
**Age (years)**	59.83 ± 12.21	55.71 ± 15.59		0.437
<50	3 (16.7)	78 (39.8)	81 (37.9)	
≥50	15 (83.3)	118 (60.2)	133 (62.1)	
**Gender (*n*)**				0.573
Female	13 (72.2)	88 (44.9)	101 (47.2)	
Male	5 (27.8)	108 (55.1)	113 (52.8)	
**Histopathology (*n*)**				
Clear cell carcinoma	15 (83.3)	151 (77.0)	166 (77.6)	0.769
Other types	3 (16.7)	45 (33.0)	48 (22.4)	

### Risk factors for IOM in patients with renal cell cancer

We examined the levels of tumor markers and clinicopathological parameters in serum samples from patients with renal cell carcinoma and analyzed differences in parameters between patients with or without IOM. The results showed that the average NSE levels of IOM group were 53.50 ± 3.53 U/l, and NIOM group were 34.64 ± 1.35 U/l. And the average Hb levels of IOM group were 91.5 ± 6.63 g/l, and NIOM group were 105.48 ± 2.01 g/l. The difference was statistically significant (*P*<0.05). There were no significant differences in other factors between the IOM and NIOM patients (*P*>0.05). The result of the binary logistic regression model suggests that NSE can be considered as an independent risk factor in the IOM group (Tables 2 and 3).

**Table 2:** differences of tumor markers between renal cancer patients with and without IOM

Clinical features	IOM group	NIOM group	*t*	*P*-value
ALP (IU/l)	337.5 ± 151.80	120.14 ± 15.66	1.424	0.172
Hb (g/l)	91.5 ± 6.63	105.48 ± 2.01	−2.014	0.045
Serum calcium (mmol/l)	2.40 ± 0.10	2.34 ± 0.03	0.629	0.530
NSE (U/ml)	53.50 ± 3.53	34.64 ± 1.35	4.100	<0.001
AFP (μg/l)	3.68 ± 0.26	4.57 ± 0.27	−0.980	0.328
CEA (μg/l)	35.38 ± 22.49	6.86 ± 2.10	1.263	0.223
CA-125 (U/ml)	41.85 ± 6.05	38.26 ± 10.46	0.104	0.917
CA-153 (U/ml)	24.48 ± 3.86	19.48 ± 0.70	1.273	0.219
CA-199 (U/ml)	18.88 ± 2.88	15.00 ± 1.13	1.017	0.311

**Table 3:** Multivariate logistic regression models analysis the risk factors of ocular metastasis from renal cancer

Factors	B	OR	OR (95% CI)	*P*
NSE	0.61	1.063	1.027–1.101	0.001
Hb	−0.17	0.983	0.963–1.004	0.121
Serum calcium	0.557	1.746	0.424–7.189	0.440
ALP	0.002	1.002	0.998–1.006	0.296
CEA	−0.007	0.993	0.967–1.021	0.628
CA-125	−0.004	0.996	0.987–1.006	0.468
CA-153	0.016	1.016	0.968–1.066	0.529
CA-199	0.012	1.012	0.979–1.046	0.490

*P*-values <0.05 represent statistical significance.

Abbreviations: B, coefficient of regression; CI, confidence interval; OR, odds ratio.

### Diagnostic value of NSE in IOM of renal cell cancer

**Figure 2:**
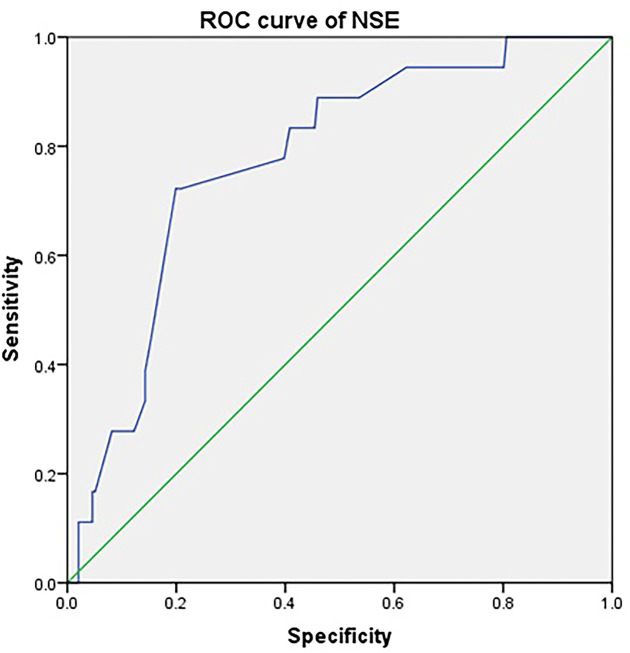
The ROC of NSE in renal cell cancer IOM patients and NIOM patients

To evaluate the value of NSE in the diagnosis and prognosis for IOM of renal cell carcinoma, we plotted its ROC curve. As shown in Table 4, the AUC of the NSE was 0.774. The specificity and sensitivity of NSE for the diagnosis of renal cell carcinoma were 72.20 and 80.10%, respectively, and the cut-off value was 49.50 U/l. The *P*-value of this index was less than 0.05, which was statistically significant (Table 4 and Figure 2).

**Table 4:** The cut-off value, sensitivity, specificity and AUC for single risk factor in predicting IOM in renal cancer patients

Factors	Cut-off value	Sensitivity (%)	Specificity (%)	AUC	*P*
NSE	49.50	72.20	80.10	0.774	<0.001

## Discussion

Renal cell carcinoma originates from renal tubular epithelial cells and is often highly malignant, accounting for more than 85% of all renal tumors. It accounts for about a second of adult malignancies, and the incidence rate is the top of the urinary system tumor, the second only to bladder cancer [10], approximately 2–3%. In the United States, there are approximately 63000 new cases of renal cell cancer each year, and approximately 14000 of them die [10]. With the progress of medical research, surgery and adjuvant radiotherapy and chemotherapy, the survival rate of patients with renal cell carcinoma has improved to a certain extent [11]. At present, in patients with incidental renal cell carcinoma, the 5-year survival rate is 85%, while that of patients with symptoms is only 53%. Patients with renal cell carcinoma without distant metastasis often have a 5-year survival rate of more than 50%, but the 5-year survival rate of patients with distant metastasis is basically less than 11% [12]. Therefore, early diagnosis of renal cell carcinoma and tumor metastasis is particularly important, early detection, early diagnosis and early treatment can make patients get a better prognosis.

The current ‘gold standard’ for the diagnosis of benign and malignant tumors is pathological detection. Although the diagnosis is objective and accurate, it needs to take the lesion tissue from the patient’s body as a specimen, which will cause some damage to the patient itself.

Imaging detection is the main tool for the detection and screening of renal cell carcinoma, but the detection cost is high, and it is easy to miss diagnosis and misdiagnosis. Some detection results can not directly and accurately show the status of the tumor. In addition, the research on IOM of renal cell carcinoma has not been carried out.

**Figure 3:**
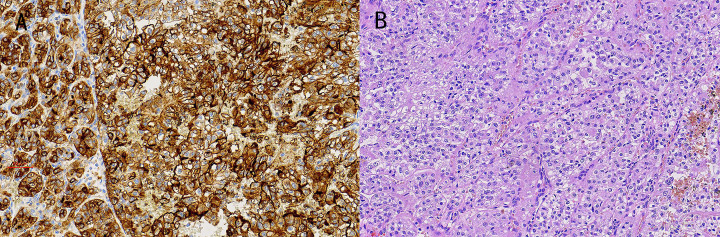
IHC images and HE staining from renal cell cancer patients with IOM (**A**) CD56 (+) (SP ×200); the tissue was collected from IOM site of renal patients. (**B**) Renal cell cancer (HE ×200).

Approximately 25–30% of renal cell carcinoma patients have cancer metastasis. Transmission is usually bloodborne, and the lung is the most frequently infected target organ, followed by bone, liver and brain. Eye position is extremely rare. The choroid is the most common site of IOM in malignant tumors. The most common primary cancers with choroidal metastasis are female breast cancer and male lung cancer [13]. In the metastatic tumors of the eye, they were mainly located in three locations: 73.6% of the patients were involved in the eyes, 12.3% were involved in the orbit and 14.1% were involved in the eyes and the orbit. At present, there is no research to prove the order of the transfer to the eye and orbit and whether there is a juxtaposition relationship [14]. In recent years, some studies have pointed out some risk factors for renal cell carcinoma metastasis (Table 5). So far, IOM has been reported in patients with non-small cell lung cancer [15], esophageal cancer [16], thyroid cancer [17], gastric adenocarcinoma [18], breast cancer [19], choriocarcinoma [20], colon cancer [21] and prostate adenocarcinoma [22] (Table 6). However, the research on IOM of renal cancer is not common. Because IOM often occurs in the late stage of renal cell carcinoma and is not common, it is difficult to detect IOM in the early stage; therefore, the early diagnosis of IOM has become a difficult problem that people hope to solve. Gómez Pascual et al. reported a 71-year-old patient with clear cell carcinoma of the kidney. The TNM stage was pT3 [23]. The patient developed IOM, which was confirmed to be nephrogenic IOM with secondary retinal detachment [23]. The interval between ocular symptoms and primary cancer symptoms in patients with IOM may be so long that the diagnostic relationship between metastasis and primary tumor becomes difficult. Tumor molecular markers are chemical substances reflecting the existence of tumors. Their expression level or activity is different from normal tissues, which helps to indicate the benign and malignant tumors, the degree of differentiation, and to further understand the appearance of tumors, cell differentiation and cancer progression. It is very helpful for cancer diagnosis, prognosis judgment and treatment guidance. In the present study, the patients with renal cell carcinoma were analyzed, and the patients without IOM and those with IOM were compared. Figure 3 shows some representative IHC images and HE-stained images, which were collected from the metastatic location (eye) of renal cell carcinoma.

**Table 5:** Risk factors leading to renal cancer metastasis

Author	Year	Risk factor
Ma et al. [35]	2015	STC1
Deml et al. [36]	2016	CD133
Low et al. [37]	2016	HSP27
Feng et al. [38]	2017	RIN1
Kou et al. [39]	2018	HMGA2

Abbreviations: HMGA2, high-mobility group AT-hook 2; HSP27, heat-shock protein 27; RIN1, Ras and Rab interactor 1; STC1, Stanniocalcin-1.

**Table 6:** Studies on the IOM from different cancers

Author	Year	Diseases with IOM	Risk factor
Singh et al. [15]	2010	Non-small cell lung cancer	ALP
Lv et al. [16]	2015	Esophageal carcinoma	PAHs
Ozpacaci et al. [17]	2012	Thyroid cancer	High TSH
Kim et al. [18]	2018	Gastric adenocarcinoma	EBV
Demirci et al. [19]	2003	Breast cancer	SNP
Hazan et al. [20]	2014	Choriocarcinoma	High HCG
Nookala et al. [21]	2016	Colon adenocarcinoma	pT1 CRC
Albadainah et al. [22]	2015	Prostatic adenocarcinoma	High-grade prostatic intraepithelial neoplasia

The table summed up studies on IOM from different types of cancer.

Abbreviations: EBV, EB virus; HCG, human chorionic gonadotropin; I PAH, polycyclic aromatic hydrocarbon; TSH, thyroid stimulating hormone.

Neuron-specific enolase (NSE) is enolase involved in glycolysis. NSE collected from the blood by non-invasive means can be used as tumor secretion or reactive secretory substances produced by the human body due to the presence of a tumor. It is helpful for the diagnosis, curative effect monitoring and prognosis evaluation of renal cell carcinoma. In patients with renal cell carcinoma, most tumor cells have two kinds of enolase isozymes: a-enolase and Y-enolase. The level of cortical Y-enolase in renal cell cancer patients is approximately 55-times than that of normal people. Moreover, 20 (49%) of 41 cases of renal cancer patients can detect elevated serum Y-enolase levels [24].

As a highly specific marker of neurons and peripheral neuroendocrine cells, elevated serum NSE level is often accompanied by malignant cell proliferation, which is the most reliable tumor marker of small cell lung cancer [25–27]. It is also considered as a marker in renal cell carcinoma, seminoma, germ cell tumor, immature teratoma, malignant melanoma, carcinoid and medullary thyroid carcinoma [28]. Mjones et al. used immunohistochemical methods to detect chromogranin A, synaptophysin, CD56, secretagogue and NSE in 178 patients with breast, lung, stomach and kidney tumors [29].

And compared with other markers. NSE was expressed in 138 cases (78%). In neuroendocrine tumors and renal clear cell carcinoma, the tumor cells with high NSE expression showed the highest staining intensity and number [29].

Kamiya et al. found that the etiology-specific survival rate of patients with tumor metastasis and high levels of NSE in the body was significantly reduced (*P*<0.05), and serum NSE as an independent variable was associated with the risk of death (*P*<0.05) [30].

Heck et al. also found the correlation between abnormal serum NSE concentration and overall survival and progression-free survival in patients with metastatic prostate cancer [31].

Muoio et al. considered that a high concentration of serum NSE represents the rapid transformation of tumor cells in the two states of mitosis and cytolysis, which may indicate that the tumor has a relatively high malignant potential [32].

Li et al. suggested that NSE level may be closely related to retinal nerve defects, and can be used as a potential biomarker of diabetic retinal neuron damage [33]. These studies suggest that serum NSE level may be used as a prognostic indicator for cancer patients. However, there is no study to explore the application value of serum NSE level in the development of IOM in patients with renal cell carcinoma. This is the first time to discuss IOM at the NSE level. The results showed that there was a significant difference in serum NSE concentration between IOM and NIOM. The serum NSE level was higher in patients with TNM stage II or IV, distant metastasis, small cell renal cell carcinoma and death during follow-up. We believe that serum NSE level is an independent risk factor associated with IOM. The critical value was 49.50 u, suggesting that serum NSE concentration > 49.50 u is a risk factor for ocular metastasis in patients with renal cell carcinoma. This helps to distinguish people with a higher risk of eye metastases. NSE is an effective index to judge tumor invasiveness and a prognostic factor for distant metastasis. Although our study has exciting findings, the present study has some limitations. First of all, the sample size of the present study is limited and the number of patients with IOM of renal cell carcinoma is small, so the data results may be biased. Second, the present study only analyzed the blood samples of patients after IOM, and did not conduct follow-up, which is part of the reason for the limitation. Thirdly, the serum NSE levels of patients with trauma, cardiovascular disease, cerebral infarction, cerebral hemorrhage and other diseases are also increased [34], so we think that dynamic monitoring of serum NSE level is more conducive to guide the treatment.

## Conclusion

The incidence of IOM of renal cell carcinoma is low, and there is no relevant research. The diagnostic index of IOM is also relatively low. In the present study, we compared the IOM and NIOM groups in renal cell carcinoma patients and found that NSE levels in the IOM group were significantly higher. After statistical analysis, NSE in the blood of patients with or without IOM had a significant differences, which can be used as a diagnostic index to predict IOM of renal cell carcinoma. Therefore, early intervention and treatment of early IOM of renal cell carcinoma can reduce the visual function and eye damage caused by tumor metastasis, and improve the quality of life of patients with renal cell carcinoma.

## Data Availability

The datasets generated during and/or analyzed during the current study are available from the corresponding author on reasonable request.

## Abbreviations

AFP: α fetoprotein
ALP: alkaline phosphatase
AUC: area under the curve
CA-125: carbohydrate antigen 125
CEA: carcinoembryonic antigen
CT: computer tomography
Hb: hemoglobin
HE: hematoxylin-eosin
IHC: immunohistochemistry
IOM: intraocular metastasis
NIOM: non-IOM
NSE: neuron-specific enolase
ROC: receiver operating curve
